# Use of 13-Valent Pneumococcal Conjugate Vaccine and 23-Valent Pneumococcal Polysaccharide Vaccine Among Children Aged 6–18 Years with Immunocompromising Conditions: Recommendations of the Advisory Committee on Immunization Practices (ACIP)

**Published:** 2013-06-28

**Authors:** Nancy M. Bennett, Tamara Pilishvili, Cynthia G. Whitney, Matthew Moore, Ryan Gierke, Aaron M. Harris

**Affiliations:** Advisory Committee on Immunization Practices Pneumococcal Work Group; Respiratory Diseases Br, Div of Bacterial Diseases, National Center for Immunization and Respiratory Diseases; EIS Officer, CDC

On February 20, 2013, the Advisory Committee on Immunization Practices (ACIP) recommended routine use of 13-valent pneumococcal conjugate vaccine (PCV13; Prevnar 13, Wyeth Pharmaceuticals, Inc., a subsidiary of Pfizer, Inc.) for children aged 6–18 years with immunocompromising conditions, functional or anatomic asplenia, cerebrospinal fluid (CSF) leaks, or cochlear implants who have not previously received PCV13. PCV13 should be administered to these children regardless of whether they received the 7-valent pneumococcal conjugate vaccine (PCV7) or the 23-valent pneumococcal polysaccharide vaccine (PPSV23). Recommendations for PPSV23 use for children in this age group remain unchanged. The evidence for the benefits and risks associated with PCV13 vaccination of children with immunocompromising conditions was evaluated using the Grading of Recommendations, Assessment, Development, and Evaluation (GRADE) framework (1). This recommendation reflects a policy change from permissive and off-label recommendation of PCV13 in the pediatric immunocompromised population to a category A recommendation (1,2). This report summarizes the evidence considered by ACIP to make this recommendation and reviews the recommendations for use of PCV13 and PPSV23 for children aged 6–18 years.

*Recommendations for routine use of vaccines in children, adolescents and adults are developed by the Advisory Committee on Immunization Practices (ACIP). ACIP is chartered as a federal advisory committee to provide expert external advice and guidance to the Director of the Centers for Disease Control and Prevention (CDC) on use of vaccines and related agents for the control of vaccine-preventable diseases in the civilian population of the United States. Recommendations for routine use of vaccines in children and adolescents are harmonized to the greatest extent possible with recommendations made by the American Academy of Pediatrics (AAP), the American Academy of Family Physicians (AAFP), and the American College of Obstetrics and Gynecology (ACOG). Recommendations for routine use of vaccines in adults are harmonized with recommendations of AAFP, ACOG, and the American College of Physicians (ACP). ACIP recommendations adopted by the CDC Director become agency guidelines on the date published in the* Morbidity and Mortality Weekly Report *(*MMWR*). Additional information regarding ACIP is available at http://www.cdc.gov/vaccines/acip.*

## Epidemiology of Pneumococcal Infection in Immunocompromised Persons and Current Recommendations

*Streptococcus pneumoniae* (pneumococcus) is a leading cause of serious infections, including sepsis and meningitis, and accounts for significant morbidity and mortality in the United States (3). PCV13 was licensed by the Food and Drug Administration (FDA) for prevention of invasive pneumococcal disease (IPD) and otitis media in infants and young children in February 2010 when it replaced the 7-valent conjugate vaccine (PCV7; Prevnar, Wyeth Pharmaceuticals, Inc.) (4). PCV13 is recommended for all children aged 2–59 months and for children aged 60–71 months with chronic medical conditions (e.g., heart disease and diabetes), immunocompromising conditions (e.g., human immunodeficiency virus [HIV]), functional or anatomic asplenia, CSF leaks, or cochlear implants. For children aged 6–18 years with immunocompromising conditions, functional or anatomic asplenia, CSF leaks, or cochlear implants, there has been a permissive and off-label ACIP recommendation (4). Vaccination with PPSV23 also is recommended for children aged 2–18 years with underlying medical conditions after completing all recommended PCV13 doses. Pneumococcal conjugate vaccines have decreased the rates of IPD directly in vaccinated children and indirectly (herd protection) in unvaccinated persons (4). Whereas overall and PCV7-type rates of IPD in HIV-infected adults decreased by 25% and 88%, respectively, rates of PCV7-type IPD in this population have remained 40 times higher than rates among healthy adults in the same age group 7 years post-PCV7 introduction for children (5). These data demonstrated that immunocompromised adults have benefitted from herd protection but still remain at high risk for disease. In June 2012, after new licensure for PCV13 in adults aged >50 years, ACIP recommended routine use of PCV13 in addition to PPSV23 for PCV13-naïve adults aged ≥19 years with immunocompromising conditions, functional or anatomic asplenia, CSF leaks, or cochlear implants (6).

During 2007–2009, the average annual incidence of IPD among children aged 6–18 years was 2.6 cases per 100,000, with 57% of IPD caused by serotypes included in PCV13 (CDC, Active Bacterial Core surveillance 2007–2009, unpublished data, 2013). Among immunocompromised children aged 6–18 years, 49% of IPD was caused by serotypes included in PCV13, and an additional 23% by serotypes included in PPSV23. Incidence rates (cases per 100,000) of PCV13-type IPD among children aged 6–18 years with hematologic malignancies were estimated at 1,282 (rate ratio [RR], compared with children without this condition, of 822; 95% confidence interval [CI] = 687–983), 197 for those with HIV infection (RR = 122; CI = 94–161), and 56 for those with sickle cell disease (SCD) (RR = 27; CI = 9–73) (Figure) (CDC, Active Bacterial Core surveillance 2007–2009, unpublished data, 2013).

## PCV13 Efficacy, Immunogenicity, and Safety Among Immunocompromised Persons

Studies of pneumococcal conjugate vaccines containing similar but fewer antigens have been conducted among persons with immunocompromising conditions. From a randomized controlled trial among HIV-infected children aged 2–45 months in South Africa, the efficacy of a 9-valent pneumococcal conjugate vaccine (PCV9) was estimated as 65% (CI = 24%–86%) against IPD and 13% (CI = −7%–29%) against radiologically confirmed pneumonia (7). Vaccine efficacy of PCV7 against PCV7-type IPD in HIV-infected adults in Malawi was estimated at 74% (CI = 30%–90%) (8). An observational study conducted in the United States among children aged ≤10 years with SCD estimated a vaccine effectiveness against IPD to be 81% (CI = 19%–96%) among those who received at least 1 dose of PCV7 (9). Although vaccine efficacy and effectiveness have been demonstrated, the duration of protection against IPD remains unknown.

In January 2013, FDA approved PCV13 in healthy children aged 6–17 years for the prevention of IPD caused by serotypes included in the vaccine. In children aged 6–9 years, vaccine effectiveness was inferred from noninferiority of immunoglobulin G (IgG) antibody responses after a single dose to IgG responses after a fourth PCV13 dose among healthy infants. For children aged 10–17 years, opsonophagocytic assay (OPA) geometric mean titers were compared with corresponding OPA titers achieved by children aged 6–9 years who were enrolled in the same study (FDA, Vaccines and Related Biological Products Advisory Committee, unpublished data, 2013). Among children with SCD aged 6–18 years, 1 dose of PCV13 elicited significant immune responses for all 13 serotypes, as measured by serotype-specific IgG concentrations (10). In a randomized trial, HIV-infected children aged 2–45 months who received 3 doses of PCV9 had significantly higher OPA titers compared with a placebo group (11). Another trial comparing PCV7 to placebo in HIV-infected children aged <2 years showed that the vaccinated children had significantly higher serotype-specific IgG titers compared with placebo (12). Data from an observational study evaluating sequential administration of 2 doses of PCV7 followed by PPSV23 in HIV-infected children aged 2–18 years showed that antibody concentrations following a single dose of PCV7 were as high or higher than following PPSV23 (13).

Current evidence supports the safety of PCV13 in children with immunocompromising conditions. An open-label, single-arm study of 158 children aged 6–18 years with SCD who previously received PPSV23 showed that 1 dose of PCV13 was safe (10). The most common adverse events reported within 7 days of 1 dose included myalgia (74.8%), fatigue (66.1%), and headache (53.6%); less common events included arthralgia (39.8%), fever (26%), vomiting (15.4%), and diarrhea (13.3%). Severe adverse events were reported among 8% of the children and included sickle cell crisis (4%), acute chest syndrome (2%), and fever (2%); no deaths were reported (10). In a PCV7 efficacy trial among HIV-infected children, the most common adverse events were severe induration, erythema, fever, and restricted leg movement; no serious adverse events were reported and no significant differences were observed in the number of adverse events between the vaccinated and unvaccinated group (12).

## PPSV23 in Immunocompromised Children

PPSV23 contains 12 of the serotypes included in PCV13, plus 11 additional serotypes, which account for 23% of IPD among immunocompromised children aged 6–18 years (CDC, Active Bacterial Core surveillance 2007–2009, unpublished data, 2013). PPSV23 currently is recommended for children aged ≥2 years with increased risk for IPD (4). Given the high burden of IPD caused by serotypes in PPSV23 but not in PCV13, broader protection might be provided through use of both PCV13 and PPSV23. There are no changes to the existing PPSV23 recommendations (4).

## ACIP Recommendations for PCV13 and PPSV23 Use in Immunocompromised Children Aged 6–18 Years

### PPSV23-naïve children

ACIP recommends that children aged 6–18 years who have not received PCV13 and are at increased risk for IPD because of anatomic or functional asplenia (including SCD), HIV infection, cochlear implant, CSF leak, or other immunocompromising conditions receive a single PCV13 dose first, followed ≥8 weeks later by a dose of PPSV23. A second PPSV23 dose is recommended 5 years after the first PPSV23 dose for children with anatomic or functional asplenia (including SCD), HIV infection, or other immunocompromising conditions (Table).

### Previous vaccination with PPSV23

Children aged 6–18 years who have not received PCV13; are at increased risk for IPD because of anatomic or functional asplenia, including SCD, HIV infection, CSF leaks, cochlear implants, or other immunocompromising conditions; and who previously received ≥1 doses of PPSV23 should be given a single PCV13 dose ≥8 weeks after the last PPSV23 dose, even if they have received PCV7. If a second PPSV23 dose is indicated, it should be given ≥5 years after the first PPSV23 dose. These children should not receive more than 2 doses of PPSV23 before age 65 years.

## Figures and Tables

**FIGURE:**
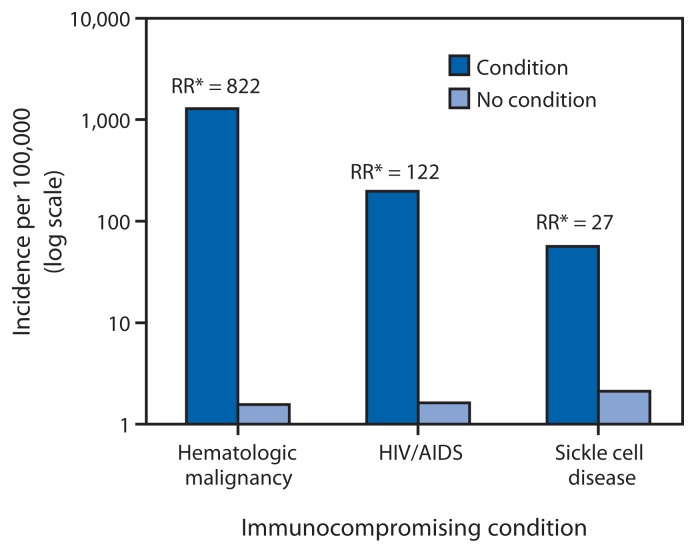
Annual average incidence of PCV13-type IPD in children aged 6–18 years, with and without selected underlying immunocompromising conditions — United States 2007–2009 **Abbreviations:** PCV13 = 13-valent pneumococcal conjugate vaccine; IPD = invasive pneumococcal disease; RR = rate ratio; HIV/AIDS = human immunodeficiency virus/acquired immunodeficiency syndrome. * RR is comparing IPD rate in children with condition listed to children without that condition.

**TABLE:** Medical conditions or other indications for administration of PCV13,* and indications for PPSV23† administration and revaccination for children aged 6–18 years§

Risk group	Underlying medical condition	PCV13	PPSV23	
	
		Recommended	Recommended	Revaccination 5 yrs after first dose
Immunocompetent persons	Chronic heart disease¶		✓	
	Chronic lung disease**		✓	
	Diabetes mellitus		✓	
	Cerebrospinal fluid leaks	✓	✓	
	Cochlear implants	✓	✓	
	Alcoholism		✓	
	Chronic liver disease		✓	
	Cigarette smoking		✓	
Persons with functional or anatomic asplenia	Sickle cell disease/other hemaglobinopathies	✓	✓	✓
	Congenital or acquired asplenia	✓	✓	✓
Immunocompromised persons	Congenital or acquired immunodeficiencies††	✓	✓	✓
	Human immunodeficiency virus infection	✓	✓	✓
	Chronic renal failure	✓	✓	✓
	Nephrotic syndrome	✓	✓	✓
	Leukemia	✓	✓	✓
	Lymphoma	✓	✓	✓
	Hodgkin disease	✓	✓	✓
	Generalized malignancy	✓	✓	✓
	Iatrogenic immunosuppression§§	✓	✓	✓
	Solid organ transplant	✓	✓	✓
	Multiple myeloma	✓	✓	✓

* 13-valent pneumococcal conjugate vaccine.

† 23-valent pneumococcal polysaccharide vaccine.

§ Children aged 2–5 years with chronic conditions (e.g., heart disease or diabetes), immunocompromising conditions (e.g., human immunodeficiency virus), functional or anatomic asplenia (including sickle cell disease), cerebrospinal fluid leaks, or cochlear implants, and who have not previously received PCV13, have been recommended to receive PCV13 since 2010.

¶ Including congestive heart failure and cardiomyopathies.

** Including chronic obstructive pulmonary disease, emphysema, and asthma.

†† Includes B-(humoral) or T-lymphocyte deficiency, complement deficiencies (particularly C1, C2, C3, and C4 deficiencies), and phagocytic disorders (excluding chronic granulomatous disease).

§§ Diseases requiring treatment with immunosuppressive drugs, including long-term systemic corticosteroids and radiation therapy.

## Footnotes

* Member roster for July 2012–June 2013 available at http://www.cdc.gov/vaccines/acip/committee/members-archive/members-07-2012-06-2013.html.
